# Enhancing access to reports of randomized trials published world-wide – the contribution of EMBASE records to the Cochrane Central Register of Controlled Trials (CENTRAL) in *The Cochrane Library*

**DOI:** 10.1186/1742-7622-5-13

**Published:** 2008-09-30

**Authors:** Carol Lefebvre, Anne Eisinga, Steve McDonald, Nina Paul

**Affiliations:** 1 UK Cochrane Centre, National Institute for Health Research, Summertown Pavilion, Middle Way, Oxford, OX2 7LG, UK; 2 Australasian Cochrane Centre, Monash Institute of Health Services Research, Monash University, Clayton, Victoria 3800, Australia

## Abstract

**Background:**

Randomized trials are essential in assessing the effects of healthcare interventions and are a key component in systematic reviews of effectiveness. Searching for reports of randomized trials in databases is problematic due to the absence of appropriate indexing terms until the 1990s and inconsistent application of these indexing terms thereafter.

**Objectives:**

The objectives of this study are to devise a search strategy for identifying reports of randomized trials in EMBASE which are not already indexed as trials in MEDLINE and to make these reports easily accessible by including them in the Cochrane Central Register of Controlled Trials (CENTRAL) in *The Cochrane Library*, with the permission of Elsevier, the publishers of EMBASE.

**Methods:**

A highly sensitive search strategy was designed for EMBASE based on free-text and thesaurus terms which occurred frequently in the titles, abstracts, EMTREE terms (or some combination of these) of reports of trials indexed in EMBASE. This search strategy was run against EMBASE from 1980 to 2005 (1974 to 2005 for four of the terms) and records retrieved by the search, which were not already indexed as randomized trials in MEDLINE, were downloaded from EMBASE, printed and read. An analysis of the language of publication was conducted for the reports of trials published in 2005 (the most recent year completed at the time of this study).

**Results:**

Twenty-two search terms were used (including nine which were later rejected due to poor cumulative precision). More than a third of a million records were downloaded and scanned and approximately 80,000 reports of trials were identified which were not already indexed as randomized trials in MEDLINE. These are now easily identifiable in CENTRAL, in *The Cochrane Library*. Cumulative sensitivity ranged from 0.1% to 60% and cumulative precision ranged from 8% to 61%. The truncated term 'random$' identified 60% of the total number of reports of trials but only 35% of the more than 130,000 records retrieved by this term were reports of trials. The language analysis for the sample year 2005 indicated that of the 18,427 reports indexed as randomized trials in MEDLINE, 959 (5%) were in languages other than English. The EMBASE search identified an additional 658 reports in languages other than English, of which the highest number were in Chinese (320).

**Conclusion:**

The results of the search to date have greatly increased access to reports of trials in EMBASE, especially in some languages other than English. The search strategy used was subjectively derived from a small 'gold standard' set of test records and was not validated in an independent test set. We intend to design an objectively-derived validated search strategy using logistic regression based on the frequency of occurrence of terms in the approximately 80,000 reports of randomized trials identified compared with the frequency of these terms across the entire EMBASE database.

## Background

Randomized trials, involving sufficient numbers of participants are essential to distinguish reliably between the effects of healthcare interventions and the effects of bias or chance. Dissemination and integration of trial results through systematic reviews of the findings provide a basis for informed decision-making about the effects of different interventions. To minimize bias due to the selective availability of data, authors of systematic reviews of healthcare interventions need to identify as many relevant randomized studies as possible to provide reliable evidence on which to base healthcare decisions [1,2].

Variations in the journals indexed in databases indicate a need to search more than one database to ensure optimal coverage of the published literature both in subject scope and language of report [3,4]. Although there is evidence that exclusion of studies in languages other than English from reviews might make no significant difference to the overall estimates of the effects of treatments [5-7], some subject areas (for example, complementary and alternative medicine) have been shown to require a more comprehensive selection of sources and unrestricted language searching in order to avoid substantial bias and increase the precision, generalizability and applicability of the findings [6,8]. EMBASE, the Excerpta Medica database published by Elsevier, complements MEDLINE/PubMed by providing greater coverage of some European publications and articles written in some languages other than English [9] as well as a broad coverage of pharmacology, psychiatry, toxicology and alternative medicine [10]. There is some evidence of added value in searching EMBASE, as well as MEDLINE, for studies for inclusion in systematic reviews, as the additional studies identified contribute to the overall findings of the review; this may be attributed in part to the greater coverage of some languages other than English in EMBASE [11,12]. The impact of the contribution may vary considerably – the overlap of EMBASE and MEDLINE has been estimated to be 10% to 87% depending on the topic under investigation [13-18] – but searchers comparing the databases have concluded that relevant studies would be missed if only MEDLINE were searched for studies in pharmacology [19] toxicology [20,21], psychiatry [3], alternative medicine [13] and other medical specialties [22-29].

Searching for reports of randomized trials presents a challenge in part because this type of study design represents only a small proportion of all the studies included in bibliographic databases. It is important, therefore, to devise a strategy which is sensitive enough to find as high a proportion as possible of all the relevant trials but specific enough not to yield vast quantities of irrelevant material, which is time-consuming, costly to evaluate and can lead to selection error.

Trial identification in databases is problematic for a number of reasons. Often the methods are not adequately described by authors in titles or abstracts and not all records in bibliographic databases have abstracts. Sensitive search strategies must, therefore, include both free text terms (used by authors in the titles and abstracts (where available) to describe their studies) and indexing terms (assigned by database indexers to describe studies) for optimal retrieval. Furthermore, suitable methodological indexing terms for randomized trials have only been introduced relatively recently and have not always been consistently applied. For example, in 1991, the United States National Library of Medicine introduced into MEDLINE the Publication Type 'Randomized Controlled Trial' as an indexing term to improve searching for trials. Despite this, a study by one of the authors (CL) [30] found that over 400 reports of randomized controlled trials indexed in the first six months of MEDLINE in 1993 were not coded with the new indexing term despite having the word random (or a variation of it such as randomized) in the title or abstract. A systematic review [31] found that it was possible to identify on average only 75% of randomized studies known to be indexed in MEDLINE. As a consequence, a highly sensitive search strategy was designed by one of the authors (CL) [31] to conduct The Cochrane Collaboration's systematic search of MEDLINE to identify reports of all definite or possible randomized or quasi-randomized trials not already indexed as randomized trials in MEDLINE, to be re-tagged in MEDLINE with the appropriate Publication Type term. Over 100,000 additional reports of randomized trials have been identified in MEDLINE back to 1966 through this electronic search, begun in 1994 by the UK Cochrane Centre and continued by the US Cochrane Center (formerly the New England Cochrane Center, which was formerly the Baltimore Cochrane Center) [32,33].

Identifying reports of trials in EMBASE has proved to be similarly problematic. Discussions began between one of the authors (CL) and Elsevier, the producers of EMBASE, in 1992, immediately after the UK Cochrane Centre opened. This led to a representative from Elsevier being invited to a workshop in January 1993, convened by the UK Cochrane Centre. It was confirmed that although the EMBASE thesaurus (EMTREE) contained terms for clinical trials in general, it had no specific term for indexing reports of randomized controlled trials. Elsevier was persuaded of the importance of accurate indexing of clinical trials and of the necessity to differentiate randomized controlled trials from other clinical trials. In September 1993, Elsevier introduced the indexing term 'Randomized Controlled Trial' in EMBASE together with the term 'Multicenter Study' and undertook to index clinical trials "even more consistently" in the future [34].

The EMBASE data structure and licensing agreements with third party vendors such as Dialog and Ovid did not, at that time, support record changes in the same way that MEDLINE did and, therefore, 're-tagging' records in EMBASE was not feasible. In addition, because The Cochrane Collaboration did not have its own register of trials at that time no further progress was made with respect to making EMBASE reports of trials available centrally within the Collaboration.

In mid-1996, however, as a result of the introduction by Elsevier of a new database platform, it became possible for them to investigate systems for updating their databases in a way that had not previously been possible. Specifically this meant that they could consider upgrading the indexing of EMBASE records by retrospectively adding their new indexing term 'Randomized Controlled Trial' to all those reports identified as such in EMBASE, thus improving retrieval in the future.

From The Cochrane Collaboration's point of view, the advent of the Cochrane Controlled Trials Register, now known as the Cochrane Central Register of Controlled Trials (CENTRAL), designed and developed by the then publishers of *The Cochrane Library*, Update Software, meant that there was now a register within the Collaboration which could provide a vehicle for making these reports accessible.

In December 1996, Elsevier requested a further meeting with one of the authors (CL) and the Managing Director of Update Software and agreed to permit the re-publication of EMBASE records in CENTRAL. Until 1996, The Cochrane Collaboration had focussed on the systematic electronic searching of MEDLINE and the systematic handsearching of general and specialized healthcare journals to facilitate access to reports of randomized trials of healthcare interventions. With the developments described above it was possible to extend this searching to include EMBASE.

It was decided that a search strategy to identify reports of randomized trials in EMBASE would be devised by two of the authors (CL and SM) [35] from an analysis of how frequently terms were used in EMBASE records to describe reports of randomized trials, that had been identified by handsearching the *BMJ *and the *Lancet *for the years 1990 and 1994 and that records of reports of randomized trials identified by using this search strategy would be published in CENTRAL.

## Objectives

The objectives of this study are:

1. To devise a search strategy, tested for sensitivity and precision, for identifying reports of randomized trials in EMBASE.

2. To identify reports of randomized trials in EMBASE that meet the Cochrane eligibility criteria [36].

3. To identify in EMBASE reports of trials not currently indexed as trials in MEDLINE, as these are already included in CENTRAL.

4. To make these reports easily accessible by including them in CENTRAL in *The Cochrane Library*, with the permission of Elsevier, the publishers of EMBASE.

## Methods

### Identifying initial search terms for testing

The Medical Subject Headings (MeSH) and Publication Type terms from the Cochrane Highly Sensitive Search Strategy for identifying reports of randomized trials in MEDLINE [31] were converted (where possible) into suitable terms from the EMBASE thesaurus, EMTREE. For example, the MeSH term 'Double-blind-Method' in MEDLINE was converted to the EMTREE term 'Double-blind Procedure'. In addition, EMTREE was examined carefully to identify additional likely candidate terms. Free-text terms were selected, including those included in the Cochrane Highly Sensitive Search Strategy for randomized trials [31] and finally, members of The Cochrane Collaboration, including those involved in devising search strategies to populate the Cochrane Review Groups' Specialized Registers of studies potentially relevant for systematic reviews, and other information specialists outside the Collaboration known to have worked on devising similar search strategies, were consulted for further suggestions.

### Creating the 'gold standard' set of EMBASE records

To test the sensitivity and precision of the search terms resulting from the above activities, a 'gold standard' set of reports of randomized controlled trials was established from the results of handsearching two general healthcare journals, the *BMJ *and the *Lancet*, for 1990 and 1994 for all reports of randomized or quasi-randomized trials. These journals had already been handsearched under another project co-ordinated by two of the authors (CL and SM) at the UK Cochrane Centre and funded by the European Union under the BIOMED Programme [37]. The intention was to create separate sensitivity and precision figures for 1990 and 1994, in order to evaluate the impact of the introduction in EMBASE in 1993 of the indexing term 'Randomized Controlled Trial' and the impact of any changes in indexing that might have arisen due to Elsevier's intention to index randomized trials "even more consistently", announced in March 1994 [34].

Two data sets were created for each of the two years. The first set, the 'gold standard' set, contained the corresponding EMBASE records for each of the reports of trials in the *BMJ *and the *Lancet *published in 1990 (n = 191) and 1994 (n = 193) found by the handsearch. The second set, the 'full EMBASE' data set, contained all *BMJ *and *Lancet *records indexed in EMBASE for the years 1990 (n = 6207) and 1994 (n = 4730).

### Testing the search terms for sensitivity and precision

Sensitivity is defined as the number of reports of randomized trials identified by the search term divided by the total number of reports of randomized trials identified, expressed as a percentage. Precision (positive predictive value) is defined as the number of reports of randomized trials identified by the search term divided by the total number of records retrieved, expressed as a percentage. Each of the search terms under consideration was searched for in the 'gold standard' set to identify how many of the reports each term identified (to calculate the sensitivity) and in the 'full EMBASE' set to identify how many records in total each term retrieved (to calculate the precision) for the year 1990 (Table 1) and 1994 (Table 2).

**Table 1:** Sensitivity and precision of terms from trial reports in the *BMJ *and *Lancet *in 1990.

	Sensitivity %	Precision %
random*	48	64
Major Clinical Study (EMTAG 0150)	42	19
Controlled Study (EMTAG 0197)	37	34
trial*	36	52
control*	34	29
study	30	22
compar*	29	24
Clinical Article (EMTAG 0152)	29	13
placebo*	25	88
blind*	23	76
doubl*	23	79
clinic*	21	16
Clinical Trial (DR)	15	53
follow* and up	15	26
Controlled Study (DE)	12	36
prospectiv*	12	23
allocat*	11	88
versus	8	45
Double-blind Procedure (DE)	7	93
Drug Comparison (DR)	7	22
vs	7	35
crossover*/cross-over*	6	100
Placebo (DE)	5	56
Major Clinical Study (DE)	5	15
Follow-up (DE)	5	13
assign*	4	73
singl*	4	16
studies	4	15
Prospective Study (DE)	3	31
Clinical Trial (DE)	3	13
volunteer*	2	25
factorial*	2	100
Comparison (DE)	1	20
mask*	1	18
tripl*	0.5	50
Clinical Study (DE)	0.5	50
longitudinal*	0.5	17
Longitudinal Study (DE)	0.5	33

The '*' denotes a free-text term with variant endings, used in the title or abstract of articles to describe the study being reported, e.g. random* will retrieve random, randomize, randomise, randomisation etc. EMTAG, DE and DR denote EMTREE descriptor terms.

**Table 2:** Sensitivity and precision of terms from trial reports in the *BMJ *and *Lancet *in 1994.

	Sensitivity %	Precision %
Controlled Study (EMTAG 0197)	83	20
Controlled Study (DE)	77	19
Clinical Trial (DE)	62	17
Major Clinical Study (DE)	58	17
Major Clinical Study (EMTAG 0150)	58	17
random*	57	59
Randomized Controlled Trial (DE)	55	69
trial*	47	48
compar*	39	34
control*	36	29
study	32	23
follow* and up	22	34
clinic*	22	15
blind*	21	71
Double-blind Procedure (DE)	19	86
placebo*	19	73
Clinical Trial (DR)	18	41
Clinical Article (EMTAG 0152)	18	10
Clinical Article (DE)	18	10
Placebo (DR)	17	56
doubl*	17	75
Follow-up (DE)	15	24
vs	14	47
Multicenter Study (DE)	12	65
assign*	11	73
prospectiv*	11	23
allocat*	10	57
versus	10	41
Drug Comparison (DR)	10	40
singl*	7	31
studies	6	14
crossover*/cross-over*	4	100
Crossover Procedure (DE)	4	89
Comparison (DE)	3	25
Phase 3 Clinical Trial (DE)	2	75
Intermethod Comparison (DE)	2	50
volunteer*	2	30
Single-blind Procedure (DE)	1	50
factorial*	1	100
longitudinal*	1	25
Prospective Study (DE)	1	18

The '*' denotes a free-text term with variant endings, used in the title or abstract of articles to describe the study being reported, e.g. random* will retrieve random, randomize, randomise, randomisation etc. EMTAG, DE and DR denote EMTREE descriptor terms

### Developing, refining and executing the search strategy

Search terms with both a precision of over 40% and a sensitivity of over 1% in either 1990 or 1994 were selected for further evaluation together with the terms follow-up, followup or follow up, volunteer$ and the descriptor term Randomization, which had not been tested in the original analysis (Table 3) [35]. The search terms included free-text terms, used in the title and/or abstract of articles to describe the study being reported and EMTREE terms assigned by the database indexers to describe the report.

**Table 3:** Search terms evaluated for systematic search of EMBASE in sequential order.

random$
factorial$
crossover$ or cross-over$
placebo$
doubl$ adj blind$
singl$ adj blind$
assign$
allocat$
volunteer$
Crossover Procedure.sh.
Double-blind Procedure.sh.
Randomized Controlled Trial.sh.
Single-blind Procedure.sh.
versus
follow-up or follow up or followup
Phase 3 Clinical Trial.sh.
Intermethod Comparison.sh.
Multicenter Study.sh.
Placebo.sh.
Randomization.sh.
trial$
vs

The '$' denotes a free-text term with variant endings, used in the title or abstract of articles to describe the study being reported, e.g. random$ will retrieve random, randomize, randomise, randomisation etc. The '.sh.' denotes an index term (EMTREE) assigned by the EMBASE indexers to describe the report.

The systematic search was conducted as a multi-file search across MEDLINE and EMBASE so that duplicate records in EMBASE indexed in MEDLINE with the Publication Type 'Randomized Controlled Trial' or 'Controlled Clinical Trial' could be removed first before downloading records unique to EMBASE for each term sequentially to be checked for eligibility.

The search terms were executed sequentially so that the incremental (cumulative) value of each term could be assessed. Cumulative sensitivity is defined as the additional number of reports of randomized trials identified by each term when searched in its position in the search sequence divided by the total number of reports of randomized trials identified, expressed as a percentage. Cumulative precision (positive predictive value) is defined as the additional number of reports of randomized trials identified by each term when searched in its position in the search sequence divided by the total number of records retrieved by that term, expressed as a percentage. Terms with low cumulative precision were rejected. Each potentially relevant record was, therefore, only retrieved once, even if it contained more than one of the terms in the search strategy. For example, a record containing the phrase 'randomized placebo controlled trial' would be identified by the first search term 'random$' but would be excluded from the set derived by the search term 'placebo$' (Table 4). The order of the terms in the sequential strategy was based on the sensitivity and precision in the 1990 search results (Table 1). The systematic search was run in two phases: (i) during 1997 and 1998, using the first four search terms, and (ii) from 1999 onwards using 22 terms, nine of which were later rejected because of low cumulative precision. The first four terms to be searched were 'random$', 'factorial', 'crossover$ or cross-over$' and 'placebo$'. Random$ and placebo$ were selected to be searched as they had relatively high sensitivity (48% and 25%, respectively) but also had relatively high precision (64% and 88%, respectively) and generated large numbers of records. Crossover$/cross-over$ and factorial$ were also selected as they were the only terms in the 1990 data set test sample that achieved 100% precision.

**Table 4:** Sequential search in EMBASE – publication years 1974–2005 (first four terms) and 1980–2005 (other terms).

	Search terms	Number of records retrieved by each search term
1	random$	130875
2	factorial$ not 1	3342
3	(crossover$ or cross-over$) not (1 or 2)	6348
4	placebo$ not (1 or 2 or 3)	36751
5	(doubl$ adj blind$) not (1 or 2 or 3 or 4)	6846
6	(singl$ adj blind$) not (1 or 2 or 3 or 4 or 5)	691
7	assign$ not (1 or 2 or 3 or 4 or 5 or 6)	22148
8	allocat$ not (1 or 2 or 3 or 4 or 5 or 6 or 7)	7097
9	volunteer$ not (1 or 2 or 3 or 4 or 5 or 6 or 7 or 8)	57510
10	Crossover Procedure.sh. not (1 or 2 or 3 or 4 or 5 or 6 or 7 or 8 or 9)	1758
11	Double-blind Procedure.sh. not (1 or 2 or 3 or 4 or 5 or 6 or 7 or 8 or 9 or 10)	5945
12	Randomized Controlled Trial.sh. not (1 or 2 or 3 or 4 or 5 or 6 or 7 or 8 or 9 or 10 or 11)	11619
13	Single-blind Procedure.sh. not (1 or 2 or 3 or 4 or 5 or 6 or 7 or 8 or 9 or 10 or 11 or 12)	612

	**TOTAL**	**291542**

Some search terms, as a result of their position in the sequence, had a cumulative precision of less than 10% in sample years (1980, 1985, 1990, 1995, and 1998) and these terms were not then used to complete the systematic search in all years (Table 5).

**Table 5:** Search results for terms rejected due to low cumulative precision in sample years in EMBASE.

Search terms in sequential order	No. of additional reports of trials identified/total no. of additional records retrieved per search term (for sample years)	Cumulative precision (%) (no. of additional reports of trials identified/total no. of additional records retrieved) (for sample years)	No. of additional reports of trials identified/total no. of additional reports of trials identified (for sample years)	Cumulative sensitivity (%) (no. of additional reports of trials identified/total no. of reports of trials identified) (for sample years)
versus	698/9163	**8**	698/3965	**18**
followup or follow up	191/8041	**2**	191/1519	**15**
Phase 3 Clinical Trial.sh.	19/398	**5**	19/2877	**0.7**
Intermethod Comparison.sh.	165/3473	**5**	165/3745	**4.4**
Multicenter Study.sh.	92/1790	**5**	92/2048	**4.4**
Placebo.sh.	178/2633	**7**	178/4364	**4**
Randomization.sh.	16/511	**3**	16/26972	**0.1**
trial$	979/11331	**9**	979/4771	**21**
vs	693/8666	**8**	693/4485	**15**

**TOTALS**	**3031/46006**	**7**		

These search terms were rejected after evaluation for the systematic search of EMBASE due to low cumulative precision (< 10%) in the sample years (1980, 1985, 1990, 1995, 1998). When terms for the systematic search were evaluated these terms were executed lower in the sequential search and therefore would be expected to have low cumulative precision as they only searched the set of records not yet identified by any of the other terms listed higher in the strategy.

### Removing duplicates

Online database providers (firstly DataStar, then Dialog and then Ovid) offering the ability to search MEDLINE and EMBASE simultaneously were used to identify and download records from EMBASE so that records which were already indexed with the Publication Type terms 'Randomized Controlled Trial' or 'Controlled Clinical Trial' in MEDLINE (and were, therefore, already included in CENTRAL) could be excluded by the EMBASE search. Initially, we used DataStar as the search interface of choice but this was limited at that time to de-duplicating 3000 records. As there were many more than 3000 records retrieved by our search sets across MEDLINE and EMBASE combined this meant that de-duplication was extremely tedious. We changed to Dialog to increase the limit to 5000 records and eventually changed to Ovid in 1999. Both DataStar and Dialog provided access to EMBASE back to 1974 but Ovid at that time only provided access back to 1980. This change meant that the remainder of the terms could not be searched back to 1974 but only back to 1980.

### Downloading and scanning

Records were downloaded and printed for the publication years 1974–2005 for the first four terms (random$, factorial$, crossover$ or cross-over$, placebo$) and 1980–2005 for the remainder (Table 4).

The title and abstract of each record was read by a trained handsearcher to identify reports of definite or possible randomized or quasi-randomized controlled trials meeting the Cochrane eligibility criteria [36]. All records were checked by a second, experienced handsearcher. Any disagreements were resolved by a third person with further reference to a clinical trialist where necessary. Records were then transferred into reference management software (ProCite) for transfer to Update Software Ltd., and latterly to John Wiley & Sons, Ltd/Wiley-Blackwell, publishers of *The Cochrane Library*, for inclusion in CENTRAL.

### Language analysis

**Figure 1:**
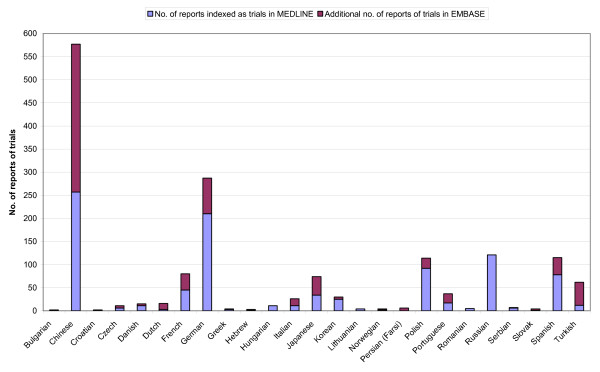
**Trial reports in MEDLINE and additional trial reports in EMBASE in 2005 in non-English languages**. This figure shows reports indexed with the Publication Type 'Randomized Controlled Trial' or 'Controlled Clinical Trial' in MEDLINE and additional reports of trials identified in EMBASE, published in 2005 in languages other than English. The reports indexed in MEDLINE include reports identified in both MEDLINE and EMBASE. The additional reports in EMBASE, therefore, indicate the added value of searching EMBASE as well as searching MEDLINE.

Reports of controlled trials in EMBASE identified in our study with the publication year 2005 were analysed according to language of publication. A comparison was made with reports of trials for the year 2005 and indexed with the Publication Type 'Randomized Controlled Trial' or 'Controlled Clinical Trial' in MEDLINE (Figure 1).

## Results

The sensitivity rankings for the search terms based on reports of trials identified from the handsearching of the *BMJ *and the *Lancet *differ from 1990 to 1994. In 1990, no EMTREE term has a sensitivity of over 50% compared with four EMTREE terms in 1994 (Tables 1 and 2). The increase in sensitivity of EMTREE terms in 1994 compared with 1990 indicates that Elsevier were indexing clinical trials more consistently in the later year.

During 1997 and 1998, 30,000 reports of trials were identified from 90,000 records downloaded from EMBASE from 1974 to 1997, using the first four search terms [9] (Table 4). Since then, a further 36,000 reports of trials have been identified from 200,000 records downloaded from EMBASE from 1980 to 2003 using 22 terms, nine of which were later rejected because of low cumulative precision (< 10%) [38] (Tables 5 and 6). During 2004 and 2005, an additional 12,000 reports of trials have been identified from 48,000 records downloaded using 13 terms (Table 6).

**Table 6:** Search results for terms selected to search EMBASE (1974–2005 first four terms; 1980–2005 other terms).

Search terms in sequential order	No. of additional reports of trials identified/total no. of additional records retrieved per search term	Cumulative precision (%) (no. of additional reports of trials identified/total no. of additional records retrieved)	Cumulative sensitivity (%) (no. of additional reports of trials identified/total no. of reports of trials identified) (n = 75,152)
**random$**	45458/130875	**35**	**60**
**factorial$**	309/3342	**9**	**0.4**
**crossover$ or cross-over$**	3304/6348	**52**	**4.4**
**placebo$**	9224/36751	**25**	**12**
**doubl$ adj blind$**	4170/6846	**61**	**6**
**singl$ adj blind$**	309/691	**45**	**0.4**
**assign$**	1709/22148	**8**	**2.3**
**allocat$**	725/7097	**10**	**1**
**volunteer$**	7496/57510	**13**	**10**
**Crossover Procedure.sh.**	266*/1758	**15**	**0.4**
**Double-blind Procedure.sh.**	524/5945	**9**	**0.7**
**Randomized Controlled Trial.sh.**	1593**/11619	**14**	**2**
**Single-blind Procedure.sh.**	65*/612	**11**	**0.1**

**TOTALS**	**75152/291542**	**26**	

When terms for the systematic search were evaluated the terms executed lower in the sequential search would be expected to have low cumulative precision as they only searched the set of records not yet identified by any of the other terms listed higher in the strategy.

* The search terms Crossover Procedure.sh. and Single-blind Procedure.sh. were introduced in 1987 and so this figure represents the total number of reports of trials identified for the publication years 1987–2005 only.

** The search term Randomized Controlled Trial.sh. was introduced in 1993 and so this figure represents the total number of reports of trials identified for the publication years 1993–2005 only.

Cumulative sensitivity ranged from 0.1% to 60% and only three terms achieved a cumulative sensitivity of 10% or more: random$ (60%), placebo$ (12%) and volunteer$ (10%) (Table 6). Cumulative precision ranged from 8% to 61% with three terms at less than 10%: factorial$ (9%), Double-blind Procedure (9%) and assign$ (8%) (Table 6).

The first term 'random$' generated the most records (130,875) of which 35% were found to be reports of controlled trials and contributed the greatest proportion of the total number of reports of trials identified (60%) (Table 6). The phrase 'doubl$ adj blind$' generated 6846 additional records, just over 60% of which were deemed to be reports of controlled trials. The phrase 'singl$ adj blind$' only generated 691 additional records, 45% of which were deemed to be reports of controlled trials. The term 'placebo$' generated 36,751 additional records, 25% of which were deemed to be reports of controlled trials and was the second largest contributor to the total number of reports of trials identified (12%). The term 'volunteer$' generated the second highest number of additional records (57,510), only 13% of which were deemed to be reports of trials. It contributed the third highest proportion of the total number of reports of trials identified (10%). The term 'assign$' generated 22,148 additional records, only 8% of which were deemed to be reports of trials. The index term 'Randomized Controlled Trial' gave a relatively low cumulative precision (14%) which partly reflects its penultimate position in the sequential strategy but also reflects the use of this term to index articles which report a randomized controlled trial but also articles which discuss randomized controlled trials from a methodological or study design aspect which would not be relevant for inclusion in CENTRAL.

In total (including results from the search terms later rejected due to low cumulative precision) approximately 350,000 records have been downloaded from EMBASE and records for all of the approximately 80,000 reports of randomized trials unique to EMBASE at the time of the searches (i.e. not also indexed as controlled trials in MEDLINE) are included in CENTRAL in *The Cochrane Library*.

The results of the language analysis indicate that for the publication year 2005 searching EMBASE did not identify any more reports of trials in Croatian, Hungarian, Lithuanian, Romanian or Russian than those already found in MEDLINE (Figure 1). Searching EMBASE also did not identify many more reports of trials in Bulgarian (1), Czech (5), Danish (4), Greek (1), Hebrew (1), Korean (5), Norwegian (2), Serbian (1) or Slovak (3) than those already found in MEDLINE. However 320 reports of trials in Chinese were identified in EMBASE in addition to the 257 already identified in MEDLINE. The reports of trials in Persian (Farsi) (6) were only identified in EMBASE. The additional reports of trials in EMBASE in Dutch (13), Italian (15) and Turkish (50) were more than those identified in MEDLINE (3, 11 and 12 respectively). Of the 18,427 reports of trials in MEDLINE with the Publication Type 'Randomized Controlled Trial' or 'Controlled Clinical Trial' published in 2005, 959 (5%) are reports in languages other than English. Of the 8464 additional reports of trials identified in EMBASE (after de-duplication of records matching reports indexed as randomized trials in MEDLINE), 658 (8%) are reports in languages other than English.

## Discussion

Projects such as this and the systematic electronic search of other bibliographic databases such as MEDLINE tend to under-identify reports of trials as there is often insufficient evidence in the title or abstract of a record to assess adequately whether it is a report of a randomized trial even if it is clearly stated in the methods section of the full journal article. In a recent study, 20 (7%) additional reports of randomized controlled trials were identified only by obtaining the full text of the article [39]. To identify these reports of trials it is necessary to handsearch the journal [1] or to read the full text of articles retrieved by terms in the search strategy or found by other means.

In addition, further reports of trials could have been identified from EMBASE by using terms with lower cumulative precision. Whilst including these terms was not considered to be feasible in the context of the project that aimed to search the whole of EMBASE, they could be considered by searchers who would be combining their study design search terms with subject- or condition-specific search terms in EMBASE and would thus retrieve considerably fewer records for consideration.

Records were de-duplicated within the host system rather than within reference management software. Any use of de-duplication facilities, either within the host system or using reference management software, may lead to over- and/or under-inclusion of records. No systematic quality control of the de-duplication process was undertaken but ad hoc viewing of the duplicate pairs seemed to indicate that the duplicates identified by the host system were valid duplicates. The eligibility criteria for including reports of trials in CENTRAL state that records should be included if they are 'definitely or possibly a report of a randomized or quasi-randomized trial'. Benefit of the doubt is, therefore, exercised where necessary. It should, however, be noted that some reports which claim to be reports of randomized trials in the title or abstract are not in fact randomized trials on the basis of further details given in the Methods section and such reports will have been included erroneously in CENTRAL as a result of this project and other similar projects where records are identified on the basis of the title and abstract only [40].

Many methodological search strategies or 'filters' have been developed in MEDLINE to make it easier to find studies of systematic reviews [41-44] and randomized controlled trials [31,36,45-62] and, more recently, in EMBASE [63,64]. The study by Wong and colleagues [63] is the only study (other than this study) of which we are aware to develop a strategy to detect 'clinically sound' treatment studies in EMBASE.

Since 1996 when the search strategy reported here was derived, methods of search strategy design have developed from subjectively derived strategies that were not performance tested [31] to subjectively derived strategies performance tested on data sets of relevant reports [58,63,65], such as this strategy, and objectively derived strategies, performance tested on such data sets [36,41,43,61]. The analysis used in 1996 to derive the terms for this EMBASE systematic search manifests a number of limitations which we intend to address in the final phase of this search strategy development. The 1996 analysis was based on a small data set (400 records of reports of trials) in two years (1990 and 1994) from two general healthcare journals (the *BMJ *and the *Lancet*) and the terms were derived subjectively.

The extent to which the derived search strategy is generalizable depends upon the sample of journals in the 'gold standard'. Boynton and colleagues have warned that the journals used in such 'gold standards' may not be representative of healthcare journals as a whole [41]. Furthermore, it has been suggested that higher impact factor journals may demand a higher standard of reporting which might bias the retrieval effectiveness of the filter when used for lower impact factor journals [43]. The *BMJ *and *Lancet*, which were used to derive the search terms in 1996, are general medical journals with medium to high impact factors, published in English.

Whether a filter developed and tested in two separate years (in this study 1990 and 1994) will give the same results for other years is likely to be affected by additions and amendments to index terms over time. Although Wilczynski and colleagues established the robustness of search strategies across publication periods 1991 and 2000 in MEDLINE [66] which led to the decision by Wong and colleagues to confine their handsearch to the year 2000 in EMBASE [63], it is not clear whether a similar robustness is present in EMBASE.

The validation of a search filter is important in assessing the effectiveness of the filter outside the set used for deriving and testing it. If the same data set is used for both purposes it has been suggested that it can introduce bias resulting in an overestimate of the effectiveness of the filter [41] because a strategy will tend to perform better on the set of records from which it was derived [43]. Wong and colleagues [63] chose not to divide their 'gold standard' into a test set (used for deriving the search filter) and a validation set (used for testing it) for their EMBASE strategies but developed and tested the strategies using the whole data set, consisting of nearly 28,000 articles. This is because their MEDLINE study [58] found that strategies developed in 60% of the data set and validated in the remaining 40% showed no statistical differences in performance.

Other initiatives, in particular the CONSORT Statement, aimed at improving the reporting of trials by authors may well have facilitated their retrieval in databases over time. CONSORT was introduced in 1996 [67] and revised in 2001 [68]. It is a checklist of 22 items and a flow diagram designed to help improve the consistency and quality of reporting of randomized controlled trials and includes a specific recommendation to identify the report as a randomized trial in the title. It has been endorsed by key healthcare journals. There is evidence that there has been an increase over time in the number of checklist items included in reports of randomized trials [69-71] which has led to reports of trials being more easily identified [59]. The CONSORT initiative was further enhanced by the publication in January 2008 of CONSORT for Abstracts which includes a checklist of 17 items designed to improve the consistency and quality of reporting of randomized trials in conference abstracts and abstracts in journal articles [72,73].

In recent years, the Centre for Reviews and Dissemination and the UK Cochrane Centre have sought to improve the objectivity of the methods used to design search strategies. They have used word frequency analysis and discriminant analysis to derive objectively, using logistic regression, the most efficient search terms and combinations of terms in titles, abstracts and index terms for particular study designs. The Group's most recent research in this area [36,61] presents a series of MEDLINE strategies with varying levels of sensitivity and precision designed to retrieve reports of randomized trials. We intend to develop this work further to complete the final phase of search strategy development to identify reports of randomized trials in EMBASE using this objective method with logistic regression and making use of the whole data set of approximately 80,000 reports of trials identified to date in the initial systematic search of EMBASE reported here.

## Conclusion

Searching EMBASE for reports of randomized trials and ensuring that they are made available in CENTRAL in *The Cochrane Library *have enhanced access to reports of trials, especially those published in languages other than English. This project has made it easier to identify approximately 80,000 reports of randomized trials by identifying the relevant records in EMBASE and including these, with the permission of Elsevier, in the Cochrane Central Register of Controlled Trials in *The Cochrane Library*. We have also identified terms that might be useful to people searching EMBASE for randomized trials in the future. However, further work remains to be done to address the limitations in the search strategy reported in this study. We intend to perform an objective analysis, using logistic regression, of the frequency of terms occurring in the approximately 80,000 reports of trials that have been identified to date, compared with their frequency across the entire EMBASE database. The results of this final analysis will be used to generate a highly sensitive search strategy for EMBASE to make reports of trials accessible to authors of reviews and others interested in basing healthcare decisions on the best available evidence.

## Key messages

EMBASE is a rich source of reports of randomized trials that are either not included in MEDLINE or not indexed as trials in MEDLINE, especially reports in some languages other than English.

Due to this project, approximately 80,000 reports of randomized trials are now more accessible through the inclusion of relevant records in the Cochrane Central Register of Controlled Trials (CENTRAL) in The Cochrane Library.

In addition to searching CENTRAL, people looking for reports of randomized trials should search EMBASE, as well as MEDLINE, for reports published in recent years that have not yet been considered for inclusion in CENTRAL.

## Abstracts in non-English languages

The abstract of this paper has been translated into the following languages by the following translators (names in brackets):

• Chinese – simplified characters (Mr. Isaac Chun-Hai Fung and Dr. Yao-Bi Zhang) [see Additional file 1]

• Chinese – traditional characters (Mr. Isaac Chun-Hai Fung and Dr. Yao-Bi Zhang) [see Additional file 2]

• French (Mr. Philip Harding-Esch) [see Additional file 3]

• Spanish (Ms. Annick Bórquez) [see Additional file 4]

## Competing interests

CL and AE work on trial identification at the UK Cochrane Centre and studies such as this may impact on their employment.

## Authors' contributions

CL conceived of the study, acquired initial project funding, negotiated publication rights with Elsevier (publishers of EMBASE), conducted the analysis which identified the original search terms, set up the original searches and co-ordinated the identification of the records in 1997, conducted the systematic search in EMBASE on backfiles, processed the resulting data for publication in CENTRAL in *The Cochrane Library*, co-authored reports of each stage of this project which have contributed to this manuscript, created the final manuscript for publication and is guarantor of the manuscript.

AE co-ordinated the identification of the records from 1999 to date, conducted the systematic search in EMBASE on backfiles and prospectively on an annual basis, reviewed and modified the search syntax annually to accommodate changes in database search structure, processed the resulting data for publication in CENTRAL in *The Cochrane Library*, co-authored a report of this stage of the project which contributed to this manuscript, wrote the first draft of this manuscript including conducting the literature search and creating the bibliography, updated the analysis of trial reports published in 1997 in languages other than English using 2005 data, and created the final manuscript for publication.

SM conducted the analysis which identified the original search terms, and co-authored a report of this stage of the project which contributed to this manuscript.

NP co-ordinated the identification of the records from 1997 to 1999, conducted the systematic search in EMBASE on backfiles, processed the resulting data for publication in CENTRAL in *The Cochrane Library*, conducted an analysis of trial reports published in 1997 in languages other than English and co-authored a report of this stage of the project which contributed to this manuscript.

All authors approved pre-publication versions of this manuscript and approved the final manuscript for publication, following peer review.

## Disclaimer

The views expressed in this study represent those of the authors and are not necessarily the views or the official policy of The Cochrane Collaboration.

## Supplementary Material

Additional file 1 Abstract in Chinese (simplified characters). Click here for file

Additional file 2 Abstract in Chinese (traditional characters). Click here for file

Additional file 3 Abstract in French. Click here for file

Additional file 4 Abstract in Spanish. Click here for file
