# Supplementary material for: Enhancing access to reports of randomized trials published world-wide – the contribution of EMBASE records to the Cochrane Central Register of Controlled Trials (CENTRAL) in The Cochrane Library
Source: Emerg Themes Epidemiol. 2008 Sep 30;5:13. doi: 10.1186/1742-7622-5-13 (PMC2586626; doi:10.1186/1742-7622-5-13)
Supplement: Additional file 1 — Abstract in Chinese (simplified characters). [file 1742-7622-5-13-S1.pdf]

Simplified Chinese / 简体中文

分析透视

增进获取世界范围随机试验报告的策略研究－EMBASE 记录对考科蓝图书馆(*The Cochrane Library*)考科蓝随机对照实验中央档案册(CENTRAL)的贡献

作者：Carol Lefebvre, Anne Eisinga, Steve McDonald 和 Nina Paul

摘要：

背景
随机试验对于评估医疗保健干预措施的效果是必不可少的，也是对其有效性所作的综合考评中的一个核心部份。在数据库中检索随机试验报告尚存在一定困难，因为在 1990 年代之前，一直缺乏适当的编纂标引术语，而其后这些标引术语的运用又不一致。

目的
本研究的目的是要建立一种检索策略以便在 EMBASE 里识别出尚未获 MEDLINE 收录的随机试验报告，以及在 EMBASE 的出版商 Elsevier 的授权下，把它们加入到考科蓝图书馆的考科蓝随机对照实验中央档案册(CENTRAL)里，从而使这些报告易于被检索到。

方法

以在 EMBASE 收录的试验报告的标题、摘要及 EMTREE 术语（或它们的一些组合）中经常出现的自由词及分类词汇为基础，设计了一种非常敏感的检索策略。用这种检索策略，在 EMBASE 里搜索由 1980 年到 2005 年的记录（其中四个术语用于搜索由 1974 年到 2005 年的记录），从索取到的记录中将未被 MEDLINE 收录的随机试验报告从 EMBASE 下载、打印并阅读。对 2005 年（本研究进行时所完成的最近年份）出版的试验报告的出版语言进行了分析。

结果

一共用了二十二个检索术语（其中九个后来因累积准确度过低而被弃用）。对超过三十多万条记录进行了下载及扫瞄，确认其中约八万条记录尚未被 MEDLINE 标引为随机试验报告。现在这些报告可以很容易地在考科蓝图书馆的 CENTRAL 中被搜索到。累积敏感度由 0.1%至 60%不等而累积准确度由 8%至 61%不等。删节术语 ‘random$’识别了索取试验报告总数的 60%，但该术语识别的十三万多个记录中只有 35%是真正的随机试验报告。以 2005 年为样本所作的出版语言分析显示在 MEDLINE 标引为随机试验的 18,427 份报告中，有 959 份(5%)是以非英语语言发表的。在 EMBASE 的搜索中，有另外 658 份被识别为用非英语语言发表的报告，其中最多为中文报告(320)。

结论

本研究结果前所未有地大幅度提高了人们对 EMBASE 里随机试验报告的访问量，特别是对非英语语言报告的访问量。所使用的检索策略是

主观地由一小组作为「金标准」的测试记录派生出来的，尚没有经独立测试验证。我们打算对一批相关术语在约八万个随机试验报告中出现的频率与其在整个 EMBASE 数据库中出现的频率进行比较，在此基础上进行逻辑回归分析，从而设计出一个客观地派生出来且通过验证的检索策略。

（中文摘要由冯俊熙及张耀璧博士翻译）
