# Supplementary material for: Enhancing access to reports of randomized trials published world-wide – the contribution of EMBASE records to the Cochrane Central Register of Controlled Trials (CENTRAL) in The Cochrane Library
Source: Emerg Themes Epidemiol. 2008 Sep 30;5:13. doi: 10.1186/1742-7622-5-13 (PMC2586626; doi:10.1186/1742-7622-5-13)
Supplement: Additional file 2 — Abstract in Chinese (traditional characters). [file 1742-7622-5-13-S2.pdf]

Traditional Chinese / 繁體中文

分析透視

增進獲取世界範圍隨機試驗報告的策略研究－EMBASE 記錄對考科藍圖書館(*The Cochrane Library*)考科藍隨機對照實驗中央檔案冊(CENTRAL)的貢獻

作者：Carol Lefebvre, Anne Eisinga, Steve McDonald 和 Nina Paul

摘要：

背景

隨機試驗對於評估醫療保健干預措施的效果是必不可少的，也是對其有效性所作的綜合考評中的一個核心部份。在數據庫中檢索隨機試驗報告尚存在一定困難，因爲在 1990 年代之前，一直缺乏適當的編纂標引術語，而其後這些標引術語的運用又不一致。

目的

本研究的目的是要建立一種檢索策略以便在 EMBASE 裡識別出尚未獲 MEDLINE 收錄的隨機試驗報告，以及在 EMBASE 的出版商 Elsevier 的授權下，把它們加入到考科藍圖書館的考科藍隨機對照實驗中央檔案冊(CENTRAL)裡，從而使這些報告易於被檢索到。

方法

以在 EMBASE 收錄的試驗報告的標題、摘要及 EMTREE 術語（或它們的一些組合）中經常出現的自由詞及分類詞彙爲基礎，設計了一種非常敏感的檢索策略。用這種檢索策略，在 EMBASE 裡搜索由 1980 年到 2005 年的記錄（其中四個術語用于搜索由 1974 年到 2005 年的記錄），從索取到的記錄中將未被 MEDLINE 收錄的隨機試驗報告從 EMBASE 下載、打印並閱讀。對 2005 年（本研究進行時所完成的最近年份）出版的試驗報告的出版語言進行了分析。

結果

一共用了二十二個檢索術語（其中九個後來因累積準確度過低而被棄用）。對超過三十多萬條記錄進行了下載及掃瞄，確認其中約八萬條記錄尚未被 MEDLINE 標引爲隨機試驗報告。現在這些報告可以很容易地在考科藍圖書館的 CENTRAL 中被搜索到。累積敏感度由 0.1%至 60%不等而累積準確度由 8%至 61%不等。刪節術語 'random$'識別了索取試驗報告總數的 60%，但該術語識別的十三萬多個記錄中只有 35%是真正的隨機試驗報告。以 2005 年爲樣本所作的出版語言分析顯示在 MEDLINE 標引爲隨機試驗的 18,427 份報告中，有 959 份(5%)是以非英語語言發表的。在 EMBASE 的搜索中，有另外 658 份被識別爲用非英語語言發表的報告，其中最多爲中文報告(320)。

結論

本研究結果前所未有地大幅度提高了人們對 EMBASE 裡隨機試驗報告的訪問量，特別是對非英語語言報告的訪問量。所使用的檢索策略是

主觀地由一小組作爲「金標準」的測試記錄派生出來的，尚沒有經獨立測試驗證。我們打算對一批相關術語在約八萬個隨機試驗報告中出現的頻率與其在整個 EMBASE 數據庫中出現的頻率進行比較，在此基礎上進行邏輯回歸分析，從而設計出一個客觀地派生出來且通過驗證的檢索策略。

（中文摘要由馮儁熙及張耀璧博士翻譯）
