# Supplementary material for: Enhancing access to reports of randomized trials published world-wide – the contribution of EMBASE records to the Cochrane Central Register of Controlled Trials (CENTRAL) in The Cochrane Library
Source: Emerg Themes Epidemiol. 2008 Sep 30;5:13. doi: 10.1186/1742-7622-5-13 (PMC2586626; doi:10.1186/1742-7622-5-13)
Supplement: Additional file 3 — Abstract in French. [file 1742-7622-5-13-S3.pdf]

French / Français

Perspectives Analytiques

Rendre les revues d’essais cliniques randomisés publiées dans le monde plus accessibles: la contribution d’enregistrements EMBASE à la base de données CENTRAL (The Cochrane Central Register of Controlled Trials) à la *Cochrane Library*.

Auteurs: Carol Lefebvre, Anne Eisinga, Steve McDonald et Nina Paul

Résumé

Contexte

Les essais randomisés sont nécessaires pour évaluer les effets des interventions des services médicaux et constituent un élément clé des revues systématiques d’efficacité. Rechercher des revues d’essais randomisés est rendu problématique par le manque de termes d’indexation appropriés avant les années 90 et par leur application variable par la suite.

Objectifs

Cette étude a pour but de concevoir une stratégie de recherche pour identifier les revues d’essais randomisés dans EMBASE qui ne sont pas déjà indexées comme des essais dans MEDLINE, et de rendre ces revues facilement accessibles en les incluant dans le Cochrane

Central Register of Controlled Trials (CENTRAL) de la *Cochrane Library* avec l'autorisation d'Elsevier, l'éditeur d'EMBASE.

Méthode

Une stratégie de recherche d'une très grande sensibilité a été conçue pour EMBASE, basée sur des termes de texte libre et de thésaurus apparaissant fréquemment dans un ou plusieurs des titres, résumés, ou termes EMTREE des revues d'essais indexées dans EMBASE. Cette stratégie de recherche a été appliquée à EMBASE pour les années 1980 à 2005 (et de 1974 à 2005 pour quatre termes). Les enregistrements extraits par cette recherche qui n'étaient pas déjà indexés comme des essais randomisés dans MEDLINE ont été téléchargés depuis EMBASE, imprimés et lus. Une analyse du langage de publication a été conduite pour les revues d'essais publiées en 2005 (l'année complète la plus récente qui était disponible lors de cette étude).

Résultats

22 termes d'interrogation ont été utilisés (dont 9 qui ont été rejetés ensuite pour cause d'une mauvaise précision cumulée). Plus de 330 000 enregistrements ont été téléchargés et scannés et environ 80 000 revues d'essais identifiées qui n'étaient pas encore indexées comme des essais randomisés dans MEDLINE. Ces revues sont aujourd'hui facilement identifiables dans le CENTRAL de la *Cochrane Library.* La sensibilité cumulée allait de 0,1% à 60% et la précision cumulée de 8% à 61%. Le terme tronqué « random$ » permettait

d’identifier 60% de la totalité des revues d’essais. Cependant, sur les 130 000 enregistrements extraits en utilisant ces termes, 31% seulement étaient des revues d’essais. L’analyse linguistique de l’année-échantillon 2005 a indiqué que sur les 18 427 rapports indexés comme des essais randomisés par MEDLINE, 959 (5%) étaient dans une langue autre que l’anglais. La recherche avec EMBASE a identifié encore 659 rapports dans des langues autres que l’anglais, dont le plus grand nombre (320) étaient en chinois.

Conclusions

Les résultats de la recherche menée à ce jour ont rendu les revues d’essais sur EMBASE beaucoup plus accessibles, surtout celles qui sont écrites dans certaines langues autres que l’anglais. La stratégie de recherche a été conçue subjectivement à partir d’un petit échantillon d’enregistrements «de référence» et n’a pas été validée sur un ensemble indépendant d’enregistrements. Nous nous proposons de concevoir une stratégie de recherche objective et validée en utilisant une régression logistique basée sur la fréquence d’ apparition de termes dans les revues d’essais randomisés qui ont été identifiées (80 000 environ) comparée à la fréquence de ces termes dans toute la base de données EMBASE.

Traduit de l’anglais par Philip Harding-Esch
