# Supplementary material for: Enhancing access to reports of randomized trials published world-wide – the contribution of EMBASE records to the Cochrane Central Register of Controlled Trials (CENTRAL) in The Cochrane Library
Source: Emerg Themes Epidemiol. 2008 Sep 30;5:13. doi: 10.1186/1742-7622-5-13 (PMC2586626; doi:10.1186/1742-7622-5-13)
Supplement: Additional file 4 — Abstract in Spanish. [file 1742-7622-5-13-S4.pdf]

Spanish / Español

Perspectiva Analítica

Mejorando el acceso a los reportes de ensayos aleatorizados publicados mundialmente- la contribución de los expedientes de EMBASE al Registro Central de Ensayos Clínicos Controlados de Cochrane (CENTRAL) en la *Biblioteca Cochrane.*

Autores: Carol Lefebvre, Anne Eisinga, Steve McDonald y Nina Paul

Resumen

Contexto
Los ensayos clínicos aleatorizados son esenciales para evaluar los efectos de las intervenciones de los servicios de salud y son un componente clave en revistas sistemáticas de eficiencia. Buscar informes de ensayos clínicos aleatorizados es problemático dada la ausencia de términos de indexación apropiados hasta los 1990's y al uso inadecuado de estos términos después de eso.

Objetivos
Este estudio se propone idear una estrategia de búsqueda para identificar informes de ensayos aleatorizados en EMBASE que no están ya indexados como ensayos en MEDLINE y de facilitar su acceso incluyéndolos en el Registro Central de Ensayos Controlados de Cochrane (CENTRAL) en la *Biblioteca Cochrane*, con el permiso de Elsevier, los editores de EMBASE.

Métodos

Una estrategia de búsqueda sumamente sensible fue diseñada para EMBASE basada en términos del texto y del tesauro que aparecieron frecuentemente en los títulos, resúmenes, términos EMTREE (o combinaciones de estos) de informes de ensayos indexados en EMBASE. Esta estrategia de búsqueda fue aplicada a los expedientes de EMBASE para los años 1980 a 2005 (1975 a 2005 para 4 de los términos). Los informes identificados en la búsqueda que no estaban ya indexados como ensayos aleatorizados en MEDLINE fueron descargados de EMBASE, impresos y leídos. Se condujo un análisis del idioma de publicación para los informes de ensayos publicados en 2005 (el año más reciente completado cuando se hizo el estudio).

Resultados

Se usaron veintidós términos de búsqueda (incluyendo nueve que fueron excluidos después por falta de precisión cumulativa). Más de un tierco de millón de informes fueron descargados y escaneados y aproximadamente 80000 informes de ensayos que no estaban ya indexados como ensayos aleatorizados en MEDLINE fueron identificados. Estos se pueden ahora identificar fácilmente en CENTRAL en la *Biblioteca Cochrane.* La sensibilidad cumulativa varió de 0.1% a 60% y la precisión cumulativa varió de 8% a 61%. El término truncado “random$” identificó al 60% del número total de informes de ensayos pero sólo 35% de más de 130000 expedientes obtenidos con este término eran informes de ensayos. El análisis de idiomas para el año de muestra 2005 indicó que de los 18 427 informes indexados como informes de ensayos aleatorizados en

MEDLINE, 959 (5%) eran en idiomas otros que el inglés. La búsqueda en EMBASE identificó 658 informes más en idiomas otros que el inglés, de los cuales la mayoría eran en chino (320).

Conclusiones

Los resultados de la búsqueda hasta la fecha han aumentado considerablemente el acceso a informes de ensayos en EMBASE, especialmente en idiomas otros que el inglés. La estrategia de búsqueda fue derivada subjetivamente a partir de un pequeño conjunto de expedientes “de referencia” y no fue validada con un conjunto independiente de expedientes. Nos proponemos diseñar una estrategia objetivamente derivada y validada usando regresión logística basada en la frecuencia de aparición de los términos en los 80000 informes de ensayos aleatorizados comparada con la frecuencia de estos términos en toda la base de datos EMBASE.

Traducido al español por Annick Bórquez
